# A Variable Temperature Synchrotron X-ray Diffraction Study of Colossal Magnetoresistant NdMnAsO_0.95_F_0.05_

**DOI:** 10.1038/srep20705

**Published:** 2016-02-15

**Authors:** E. J. Wildman, A. C. Mclaughlin

**Affiliations:** 1 The Chemistry Department, University of Aberdeen, Meston Walk, Aberdeen, AB24 3UE, Scotland

## Abstract

The recent discovery of high temperature superconductivity in Fe arsenides has invigorated research into transition metal pnictides. Colossal magnetoresistance (CMR) has recently been reported for NdMnAsO1-xFx for x = 0.05–0.08, with a maximum magnetoresistance achieved at low temperature (MR_9T_(3 K)) = −95%). This appears to be a novel mechanism of CMR, which is as a result of a second order phase transition in field from an insulating antiferromagnet to a semiconducting paramagnet. Here we report a variable temperature synchrotron X-ray powder diffraction study of the CMR oxypnictide NdMnAsO_0.95_F_0.05_ between 4 K–290 K. An excellent fit to the tetragonal unit cell with space group P4/*nmm* is obtained over the entire temperature range, with no change in crystal structure detected down to 4 K. A coupling of the lattice and magnetic order is observed, where subtle discontinuities in the temperature variation of *a* and the *c/a* ratio are apparent as the Nd spins order antiferromagnetically and the Mn moments reorient into the basal plane at *T*_*SR*._ The results suggest that very small changes in lattice parameters effect the coupling between lattice, electronic and magnetic degrees of freedom.

The discovery of high temperature superconductivity in the quaternary oxypnictide LaFeAsO^1^ reinvigorated research into transition metal oxypnictides. Superconductivity may be induced in 1111-type pnictides upon substituting oxygen with fluorine^2^,^3^,^4^, creating oxygen vacancies^5^ or by replacing the rare earth with Th^4+^ 6, with a current maximum *T*_*c*_ of 56.3 K achieved in Gd_1−x_Th_x_FeAsO. The LaFeAsO parent compound crystallizes with a tetragonal unit cell in the P4/*nmm* space group, with a conducting Fe_2_As_2_ layer situated between insulating La_2_O_2_ planes^7^. A structural distortion from tetragonal to orthorhombic symmetry (space group *Cmma)* occurs upon cooling with antiferromagnetic (AFM) ordering below *T*_*s*_^8^,^9^. The superconducting phase appears when the structural and magnetic ground state are suppressed (e.g. by chemical doping^10^) and can also be induced in some non-doped systems by the application of external pressure^11^,^12^,^13^. An empirical relation exists between the distortion of As-Fe-As bond angles and the onset temperature for superconductivity (*T*_*c*_), as maximum values are achieved when regular tetrahedra are formed in the FeAs_4_ lattice^14^, indicating a clear relationship between the crystal structure and superconductivity.

While the manganese analogues (*Ln*MnAsO, *Ln* = La, Nd) are not superconducting, they have been shown to exhibit sizeable magnetoresistance (MR) between ∼150 K–380 K, with MR values of up to −24% observed at 200 K for LaMnAsO^15^,^16^. Magnetoresistance is defined as the change of electrical resistivity, ρ, in an applied magnetic field, *H*, so that MR = (*ρ*(*H*)−*ρ*(0))/*ρ*(0); materials exhibiting this effect are important for memory device and magnetic sensor applications. Further studies revealed that colossal magnetoresistance (CMR) is observed upon substituting F^−^ for O^2−^ in NdMnAsO_1−*x*_F_*x*_ (x = 0.05–0.08). A maximum MR is achieved in NdMnAsO_0.95_F_0.05_ at low temperature (MR_9T_(3 K)) = −95%)^17^.

In contrast to the Fe superconductors no structural distortion is apparent in NdMnAsO_0.95_F_0.05_ down to 4 K^17^; yet, neutron diffraction studies show that several magnetic transitions exist. Antiferromagnetic ordering of the Mn^2+^ spins occurs at 356 K with moments aligned parallel to *c*, followed by ordering of the rare earth at 23 K  K where Nd^3+^ spins order antiferromagnetically with moments aligned parallel to the basal plane. At the same time a spin reorientation of the Mn spins occurs, as they rotate from their original alignment along the *c* axis into the *ab* plane so that by *T*_*SR*_ = 20 K the Mn spins are also aligned parallel to the basal plane. MR is observed below 75 K and increases further below *T*_*SR*_. It has been proposed that the CMR in NdMnAsO_0.95_F_0.05_ arises due to a hidden order parameter, resulting in competition between an AFM insulating phase and a paramagnetic semiconductor upon application of a magnetic field^17^. Furthermore, recent high pressure neutron diffraction studies revealed that the AFM ordering of Mn spins in NdMnAsO_0.95_F_0.05_ are robust up to pressures of 8.59 GPa and T_Mn_ is enhanced from 360–383 K upon applying an external pressure of 4.97 GPa^18^. NdMnAsO_0.95_F_0.05_ is however shown to violate Bloch’s rule which would suggest that NdMnAsO_0.95_F_0.05_ is on the verge of a localised to itinerant transition^18^.

Changing the rare earth from Nd to Pr in *Ln*MnAsO_0.95_F_0.05_ has a dramatic effect on the structural, magnetic and electronic properties of the manganese materials. Variable temperature synchrotron X-ray results describe a structural transition from tetragonal to orthorhombic symmetry with space group *Pmmn* below 35 K in PrMnAsO_0.95_F_0.05_^19^. The distortion is the result of ferromultipolar ordering of Pr spins and is associated with a sizeable negative MR (MR_7T_ (12 K) = −13.4%), instead of the CMR observed in the Nd analogue^17^.

In order to further investigate the relationship between the crystal structure and electronic and magnetic properties of the CMR material NdMnAsO_0.95_F_0.05_, we have performed a variable temperature synchrotron X-ray diffraction study between 4 K and 290 K. The results demonstrate that there is no change in crystal structure within the temperature range studied in contrast to PrMnAsO_0.95_F_0.05_ and the superconducting Fe analogues. However, subtle discontinuities in the *a* lattice parameter and *c/a* ratio are observed at *T*_*SR*_.

## Results and Discussion

**Figure 1:**
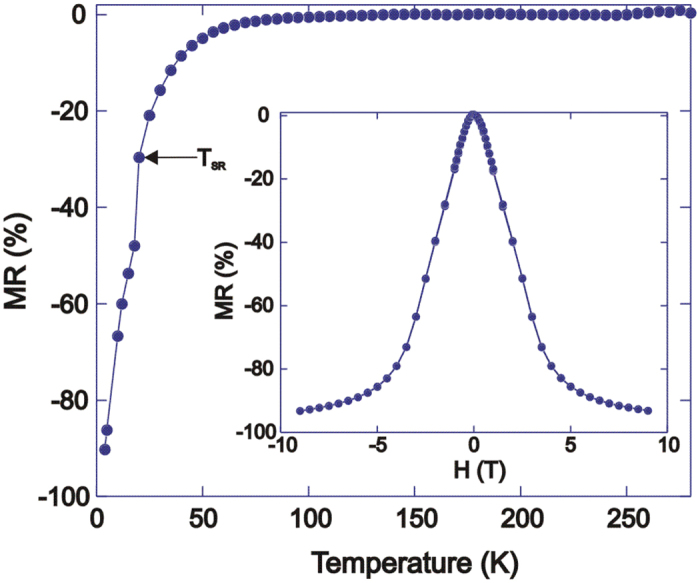
Magnetoresistance data as a function of temperature and field for NdMnAsO_0.95_F_0.05_. A MR of −90% at 4 K, 7 T is observed. The inset shows the variation of MR with field at 4 K.

The temperature dependence of the 7 T magnetoresistance of NdMnAsO_0.95_F_0.05_ is displayed in Fig. 1. As reported previously MR is observed below ∼80 K and its magnitude increases exponentially upon cooling^17^. The magnitude of the MR rises sharply below *T*_*SR*_ so that at 4 K MR_7T_ = −90%. The field variation of the MR is also displayed in Fig. 1 and reproduces previous results^17^.

The variable temperature synchrotron X-ray powder diffraction data were analysed using the Rietveld refinement method^20^ and the GSAS programme^21^ to determine the crystal structure. The backgrounds were fitted using linear interpolation and the peak shapes were modelled using a pseudo–Voigt function. A minor impurity phase MnAs is observed and was modelled giving a volume fraction of 1.05%.

**Figure 2:**
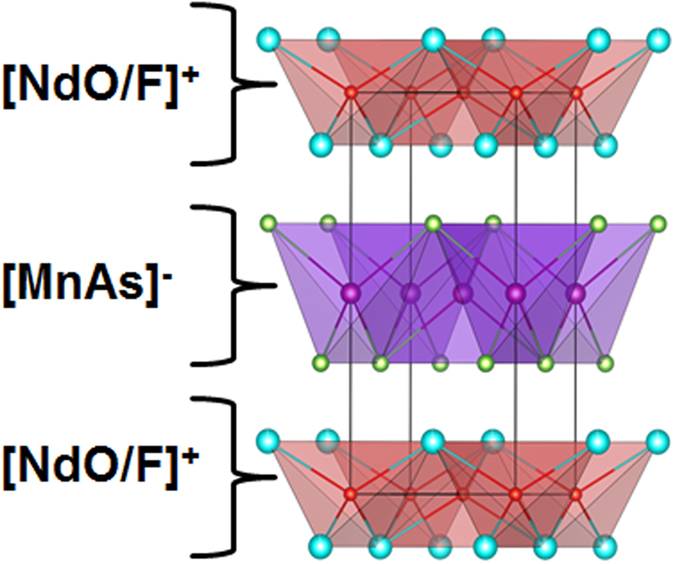
Crystal Structure of NdMnAsO_0.95_F_0.05_. The (NdO/F)^+^ and (MnAs)^−^ slabs are labelled.

**Figure 3:**
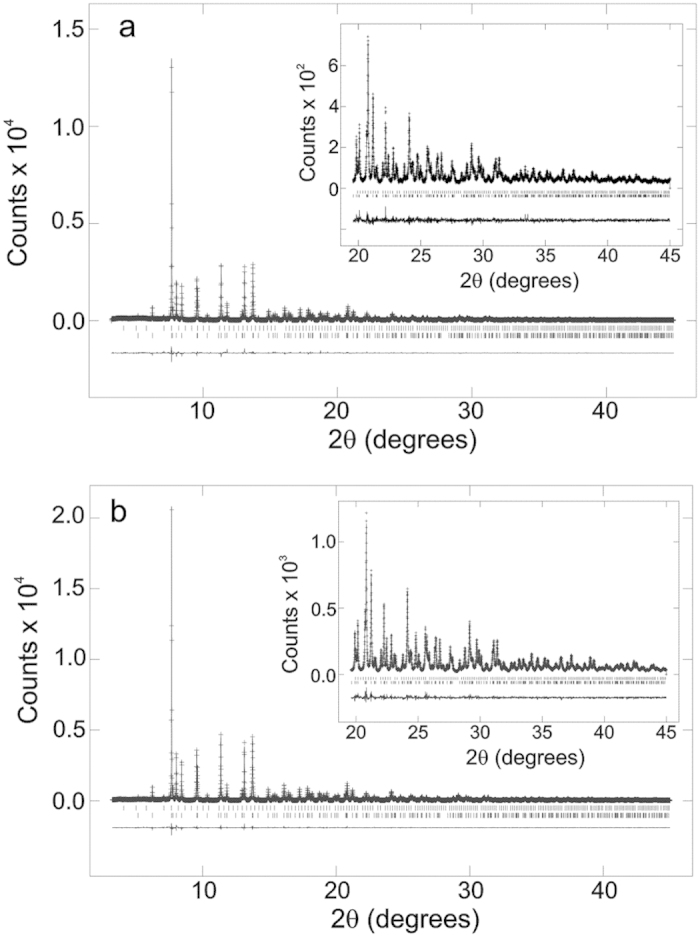
Rietveld refinement fits to the 290 K and 4 K ID31 synchrotron X-ray powder diffraction patterns of NdMnAsO_0.95_F_0.05._ The 290 K and 4 K Rietveld refinement fits are shown in (a) and (b) respectively. Tick marks represent reflection positions for NdMnAsO_0.95_F_0.05_ and MnAs minor impurity phase from bottom to top respectively.

The Rietveld refinement of high resolution synchrotron X-ray powder diffraction data collected between 4 and 290 K confirmed that NdMnAsO_0.95_F_0.05_ crystallises at room temperature with the expected ZrCuSiAs-type tetragonal structure of space group *P*4*/nmm* (Fig. 2) where insulating layers of ionic (NdO/F)^+^ are embedded between layers of tetrahedral (MnAs)^−^. An excellent fit to this space group is obtained at all temperatures (Fig. 3). There is no evidence of peak splitting or superstructure peaks to suggest a change in symmetry upon cooling. The refined values for lattice constants, atomic parameters, selected bond lengths and angles with corresponding agreement indices for the respective variable temperature fits to the data are found in Table 1. There is no evidence of cation or As/O anion disorder. The Nd, Mn and As occupancies refined to within ±1% of the full occupancy and were fixed at 1.0. The O and F occupancies were fixed at 0.95 and 0.05 respectively.

**Figure 4:**
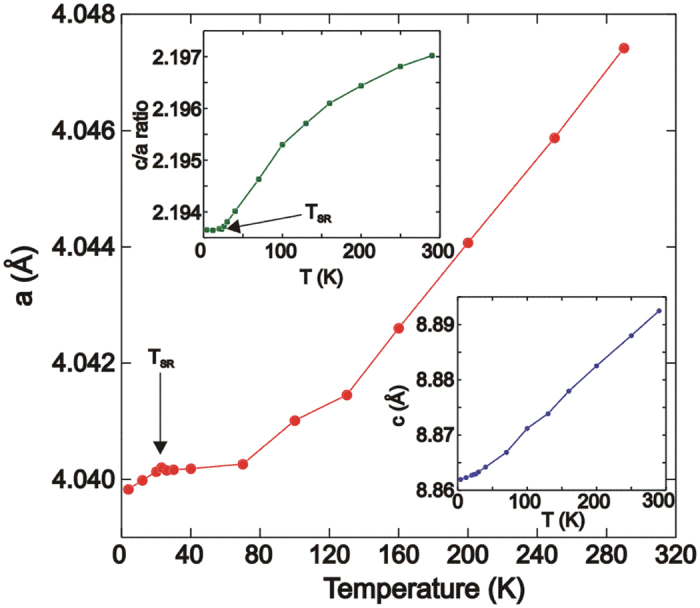
Temperature variation of refined cell parameters and *c/a* ratio. A subtle discontinuity is observed at *T*_*SR*_ in the temperature variation of *a* and *c/a*. This anomaly is not observed in the temperature dependence of the *c* cell parameter.

**Figure 5:**
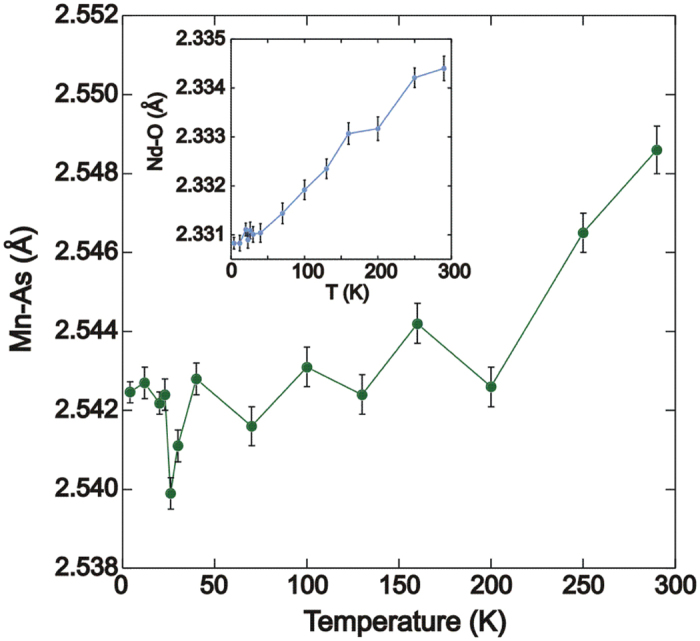
The temperature dependence of the Mn-As and Nd-O bond lengths. A reduction in both bond lengths is observed upon cooling to 4 K.

The temperature dependence of the cell parameters are shown in Fig. 4. A subtle anomaly is observed in the temperature variation of the *a* cell parameter where a change in slope is detected at 23 K (*T*_*SR*_). This discontinuity is not present in the temperature variation of the *c* cell parameter, which exhibits a normal thermal expansion (Fig. 4 (inset)) but is apparent in the *c*/*a* ratio (Fig. 4 (inset)). It is also not present in any of the bond lengths or angles upon cooling to 4 K (Table 1). The variation of the Mn-As and Nd-O bond lengths with temperature are shown in Fig. 5. Both bond lengths decrease upon cooling. The As-Mn-As and Nd-O-Nd bond angles do not change significantly with temperature (Table 1).

The subtle anomaly at *T*_*SR*_ in the *a* cell parameter is not evident in the parent compound NdMnAsO^22^. However the c/a ratio does evidence a change in slope at *T*_*SR*_ evidencing a weak coupling between the lattice and the magnetic order^22^. It appears that a stronger coupling of the lattice and magnetic order is present in NdMnAsO_0.95_F_0.05_, where changes in *a* and the *c/a* ratio are much more apparent at T_*SR*._ This stronger coupling could be a result of the lattice contraction upon substitution of F^−^ for O^2−^ (*a* and *c* shrink from 4.0503(1) and 8.9150(4) to 4.0500(1) and 8.9040(4) upon increasing *x* from 0 to 0.05 in NdMnAsO_1−*x*_F_*x*_^17^). In *Ln*MnAsO the Dzyaloshinskii-Moriya (DM) and biquadratic (BQ) exchanges between the *Ln*^3+^ and Mn are strong and control the spin reorientation transition^23^; the BQ exchange dominates in NdMnAsO. In principle the smaller unit cell in NdMnAsO_0.95_F_0.05_ could enhance magnetic exchange between Nd^3+^ and Mn^2+^ ions which in turn will then augment the spin-lattice coupling at T_SR_.

The electronic properties of NdMnAsO and fluorine doped samples, NdMnAsO_1−*x*_F_*x*_, are also very different. Above 90 K the electronic behaviour of NdMnAsO_0.95_F_0.05_ is dominated by thermally activated charge carriers across a band gap so that *ρ* = *ρ*_*0*_ exp (*E*_*g*_/2*kT*) (*E*_g_** = 23 meV)^17^. The temperature variation of the resistivity can be modelled by three-dimensional variable range hopping (VRH)^24^ of the carriers below 85 K (the resistivity, *ρ*, is defined as *ρ* = *ρ*_*0*_ exp (*T*_*0*_*/T*)^0.25^). In the variable range hopping mechanism, a localised electron can only move from one localised site to another by phonon assisted hopping, which is a combined thermally active quantum tunnelling process. An electron will only tunnel to another site if the thermal activation energy required for the hop is reduced. Below *T*_*SR*_, in NdMnAsO_1−*x*_F_*x*_ (*x* = 0.05–0.08), the spin reorientation of the Mn spins from aligning along *c* to aligning parallel to *a* precipitates an electronic transition from three dimensional Mott variable range hopping (VRH) to Efros Shklovskii (ES) VRH^25^. This signifies that the reorientation of Mn spins into the basal plane results in enhanced Coulomb correlations between localized electrons^17^, which results in much higher resistivity below *T*_*SR*_. This transition is not observed in the parent compound^22^. The transition to ES VRH in NdMnAsO_1−*x*_F_*x*_ (*x* = 0.05–0.08) is crucial for the appearance of CMR in F^−^ doped materials, as the CMR arises due to a reduction in Coulomb correlations upon application of a magnetic field^17^. A transition from an insulating antiferromagnet to a semiconducting paramagnet is observed upon applying a magnetic field, which results in an electronic transition from ES VRH to Mott VRH.

It is highly likely that the stronger lattice response to the spin reorientation transition in NdMnAsO_0.95_F_0.05_ precipitates the electronic transition to ES VRH, as the *a* cell parameter suddenly contracts below T_*SR*_ and Coulomb correlations are enhanced. It has previously been shown that the electronic structure of LnFeAsO systems strongly depends on small changes in interatomic distances^26^. It would appear that the same may be true for the 1111 Mn^2+^ analogues and further studies of the electronic structure are warranted.

In summary we have investigated the temperature dependence of the crystal structure of NdMnAsO_0.95_F_0.05_. There is no evidence of a change in crystal symmetry upon cooling but there is a subtle lattice anomaly at *T*_*SR*_ in the temperature variation of the *a* cell parameter and also the *c/a* ratio. We propose that this coupling between the lattice and magnetic order results in the electronic transition to ES VRH below *T*_*SR*_ so that a coupling between lattice, electronic and magnetic degrees of freedom is evident in the CMR material NdMnAsO_0.95_F_0.05_.

## Methods

### Synthesis

A polycrystalline sample of NdMnAsO_0.95_F_0.05_ was synthesised via a two-step solid-state reaction method. Initially, the NdAs precursor was obtained by the reaction of Nd pieces (Aldrich 99.9%) and As (Alfa Aesar 99.999%) at 900 °C for 24 h in an evacuated, sealed quartz tube. The resulting precursor was then reacted with stoichiometric amounts of MnO_2_, Mn and MnF_2_ (Aldrich 99.99%), all powders were ground in an inert atmosphere and pressed into pellets of 10 mm diameter. The pellets were placed into a Ta crucible and sintered at 1150 °C for 48 h, again in a quartz tube sealed under vacuum.

### Physical Measurements:

The temperature dependence of the electrical resistance was recorded using a Quantum Design physical property measurement system (PPMS) between 4 and 280 K in magnetic fields of 0 T and 7 T. The field dependence of the electrical resistance was recorded in magnetic fields of ±9 T.

### Structural Characterisation

High resolution synchrotron X-ray powder diffraction patterns of NdMnAsO_0.95_F_0.05_ were recorded on the ID31 beamline at the ESRF, Grenoble, France at selected temperatures between 4 K and 290 K with a wavelength of 0.3999 Å. The powder sample was inserted into a 0.5 mm diameter borosilicate glass capillary and spun at ~1 Hz to improve the powder averaging of the crystallites. Diffraction patterns were collected over the angular range 2–45° 2θ and rebinned to a constant step size of 0.002° for each scan.

## Additional Information

**How to cite this article**: Wildman, E. J. and Mclaughlin, A. C. A Variable Temperature Synchrotron X-ray Diffraction Study of Colossal Magnetoresistant NdMnAsO_0.95_F_0.05_. *Sci. Rep.* **6**, 20705; doi: 10.1038/srep20705 (2016).

## Figures and Tables

**Table 1:**
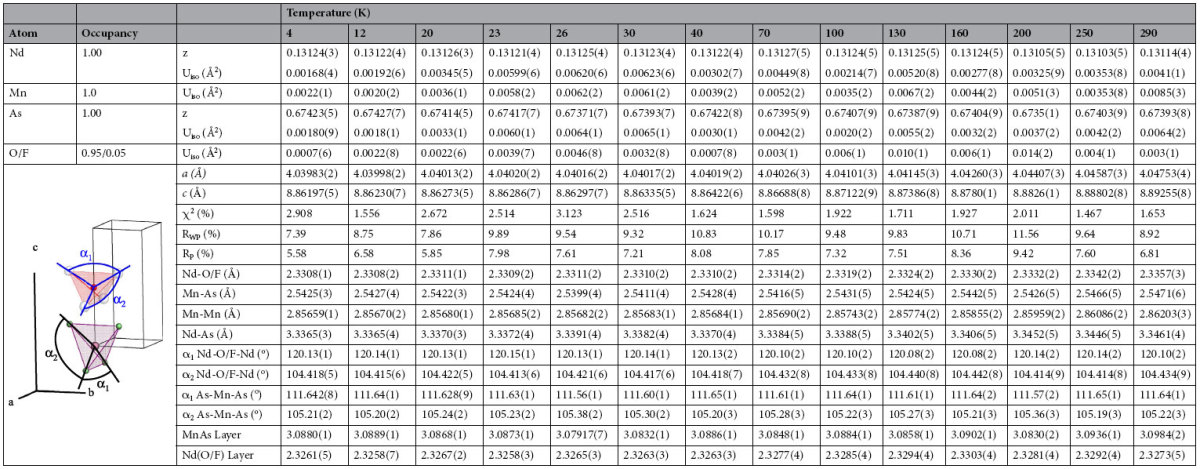
Refined cell parameters, agreement factors, atomic parameters and selected bond lengths and angles for NdMnAsO_0.95_F_0.05_ from Rietveld fits against X-ray synchrotron data at various temperatures.

Nd and As are at 2c (1/4, 1/4, z), Mn at 2b (3/4, 1/4, 1/2) and O,F at 2a (3/4, 1/4, 0).
